# Appropriateness of Antiplatelet Therapy and Proton Pump Inhibitor Prescribing in End-Stage Kidney Disease: A Retrospective Quality Investigation Study

**DOI:** 10.1177/20543581241312618

**Published:** 2025-01-06

**Authors:** Michael Che, Sumaiya Ahmed, Ryan Chan, Ayub Akbari, Deborah Zimmerman

**Affiliations:** 1 Division of Nephrology, Department of Medicine, Faculty of Medicine, University of Toronto, ON, Canada; 2 Division of Nephrology, Department of Medicine, Ottawa Hospital, University of Ottawa, ON, Canada

**Keywords:** bleeding, anticoagulation, hemodialysis, quality improvement, patient safety

## Abstract

**Background:**

Patients with end-stage kidney disease (ESKD) have high rates of gastrointestinal bleeding due to several risk factors including platelet dysfunction, comorbid illness, and use of antiplatelet medications. Proton pump inhibitors (PPIs) reduce gastrointestinal bleeding and are recommended for high-risk patients such as those prescribed dual antiplatelet therapy (DAPT). Whether inappropriate duration of DAPT therapy and/or lack of appropriate PPI use contribute to the known elevated risk of gastrointestinal bleeding in hemodialysis patients is not known.

**Objectives:**

To determine whether patients with ESKD are appropriately prescribed DAPT and PPI therapy.

**Design:**

Retrospective cross-sectional, quality investigation.

**Setting:**

Satellite hemodialysis unit of a tertiary care center in Ontario, Canada.

**Patients:**

All patients with ESKD treated at a satellite hemodialysis unit of a tertiary care hospital.

**Measurements:**

Number of patients prescribed antiplatelets, PPIs, anticoagulants, non-steroidal anti-inflammatory drugs (NSAIDs), and corticosteroids; indications for aforementioned medications.

**Methods:**

A chart review was performed to elucidate patients’ medical history and pertinent medications. Patients’ indications for PPI and DAPT were extracted from their electronic medical records.

**Results:**

Out of 88 patients with ESKD treated with hemodialysis, 44 were on antiplatelet therapy (4 on DAPT), 1 on NSAID, 12 on corticosteroids, 7 on oral anticoagulants, 2 on histamine H2-receptor antagonists, and 39 on PPIs. Fourteen percent of PPI users had absolute indication for therapy. One patient in whom PPI therapy was indicated was not prescribed one. Out of 4 patients on DAPT, 3 had current indications for DAPT, whereas 1 had a prior indication.

**Limitations:**

Single-center study; medication lists obtained from electronic medical records not confirmed by patient interview.

**Conclusions:**

At the time of this study, 3% of patients with ESKD treated with hemodialysis had a current indication for DAPT. One patient prescribed DAPT no longer met indication for therapy and was reduced to single antiplatelet therapy. Only one patient with an absolute indication for PPI therapy had not been prescribed one. Overall, it appears that prescribing patterns of DAPT and PPI at our center are unlikely to be a major contributor to the known increased risk of gastrointestinal bleeding in patients treated with hemodialysis. However, this may not be true of all units; ensuring regular medication reviews are undertaken may enhance appropriate prescribing.

## Abrégé

**Contexte:**

Les patients atteints d’insuffisance rénale terminale (IRT) présentent des taux élevés de saignements gastro-intestinaux en raison de plusieurs facteurs de risque, notamment d’une dysfonction plaquettaire, de comorbidités et de l’utilisation de médicaments antiplaquettaires. Les inhibiteurs de la pompe à protons (IPP) réduisent les saignements gastro-intestinaux et sont recommandés chez les patients à haut risque comme ceux qui ont reçu une bithérapie antiplaquettaire (DAPT). On ignore si le risque élevé connu de saignements gastro-intestinaux chez les patients sous hémodialyse est influencé par la durée inappropriée du traitement par DAPT et/ou une utilisation inappropriée des IPP.

**Objectifs:**

Déterminer si les patients atteints d’IRT reçoivent le traitement approprié avec la DAPT et les IPP.

**Conception:**

Étude rétrospective transversale évaluant la qualité.

**Cadre:**

L’unité d’hémodialyse satellite d’un centre de soins tertiaires en Ontario (Canada)

**Sujets:**

Tous les patients atteints d’IRT traités dans l’unité d’hémodialyse satellite d’un hôpital de soins tertiaires.

**Mesures:**

Le nombre de patients ayant reçu des antiplaquettaires, des IPP, des anticoagulants, des anti-inflammatoires non stéroïdiens (AINS) et des corticostéroïdes, ainsi que les indications pour les médicaments susmentionnés.

**Méthodologie:**

Un examen des dossiers a été réalisé afin de connaître les antécédents médicaux des patients et les médicaments pertinents. Les indications pour la DAPT et la prise d’IPP ont été extraites des dossiers médicaux électroniques des patients.

**Résultats:**

Sur les 88 patients atteints d’IRT traités par hémodialyse, 44 suivaient un traitement antiplaquettaire (4 sous DAPT), 1 prenait des AINS, 12 des corticostéroïdes, 7 des anticoagulants oraux, 2 des antagonistes des récepteurs H2 de l’histamine et 39 des IPP. Quatorze pour cent des utilisateurs d’IPP avaient une indication absolue pour le traitement. Un des patients pour qui un traitement par IPP était indiqué n’avait pas reçu de prescription. Des 4 patients sous DAPT, 3 avaient des indications actuelles pour une DAPT alors que le quatrième avait une indication antérieure.

**Limites:**

Étude monocentrique; les listes de médicaments obtenues à partir des dossiers médicaux électroniques n’ont pas été confirmées par l’entretien avec le patient.

**Conclusion:**

Au moment de cette étude, 3 % des patients atteints d’IRT traités par hémodialyse avaient une indication actuelle pour une DAPT. Un patient ayant reçu une DAPT ne répondait plus à l’indication du traitement et le traitement a été modifié pour une monothérapie antiplaquettaire. Un seul patient avait une indication absolue pour le traitement par IPP, mais n’avait pas reçu de prescription. Dans l’ensemble, il apparait que la prescription d’une DAPT et d’IPP dans notre centre est peu susceptible de contribuer de façon importante à l’augmentation connue du risque de saignement gastro-intestinal chez les patients traités par hémodialyse. Cela pourrait ne pas être le cas pour toutes les unités d’hémodialyse. S’assurer d’examiner les médicaments sur une base régulière pourrait améliorer l’adéquation de la prescription.

## Background

The risk of hospitalization for an upper gastrointestinal (GI) bleed in patients with end-stage kidney disease (ESKD) ranges from 21 to 328/1000 patient years depending on study design, definitions, ascertainment of outcome, and concurrent use of anti-thrombotic medications.^1-3^ In a more recent study from the United States, 406 836 incident dialysis patients were followed for 832 131 patient years. Approximately 1 in 5 patients had at least one GI bleed hospitalization event; the majority of which were upper GI bleeds.^3^ The overall risk appeared to be increasing over time and was greater with increasing age, in women and blacks versus whites but lower in patients treated with peritoneal dialysis compared with hemodialysis (HD).^3^ Clinically relevant non-major GI bleeds and GI bleeds requiring transfusions but managed conservatively without hospitalization or endoscopy were not captured.

**Figure 1.**
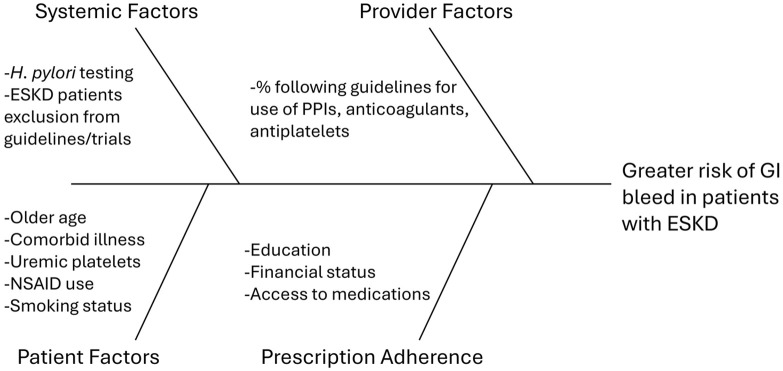
Factors contributing to gastrointestinal bleed risk in end-stage kidney disease. *Note. H. pylori* = *Helicobacter pylori*; ESKD = end-stage kidney disease; PPI = proton pump inhibitor; NSAID = non-steroidal anti-inflammatory drug; GI = gastrointestinal.

Importantly, from a patient perspective, the need for a blood transfusion increases the risk for allosensitization, prolongs renal transplant wait times, and results in higher rates of graft rejection and lower rates of graft survival.^4^ The factors predisposing to this increased bleeding risk are multifactorial (Figure 1). End-stage kidney disease is associated with anemia, platelet dysfunction, and impaired interactions between platelets and the vessel wall.^5^ *Helicobacter pylori*, a known risk factor for upper GI bleeds, affects patients with ESKD at a prevalence of 44%, comparable to the high rate in the general population globally.^6^ In addition, patients typically receive anticoagulation 3 times per week during the HD treatment. Furthermore, due to the risk of cardiovascular disease, many patients with ESKD will be treated with single or dual antiplatelet therapy (DAPT).

Proton pump inhibitors (PPIs) tend to be overprescribed and have side effects such as hypomagnesemia, *Clostridium difficile* infection, pneumonia, Vitamin B12 deficiency, and fractures, but they do serve an important role in the prevention of upper GI bleeding in high-risk patients taking non-steroidal anti-inflammatory drugs (NSAIDs), aspirin, or DAPT.^7,8^ A study conducted by Ray and colleagues found that PPIs decreased the risk of upper GI bleeding in patients on clopidogrel by 50% and decreased the risk of GI bleeding by 2.8% per year in patients with more than 3 risk factors including age 65 years or older, prior history of hospitalization for upper GI disease or bleeding, recent use of anticoagulants, current use of other medications that increase bleeding risk, and any hospital discharge in the past year.^9^

In our previous quality assurance project, more than 50% of patients were being treated with aspirin and/or other antiplatelet agent.^10^ During that study, we did not assess the appropriateness of DAPT.^10^ We also did not verify if there were patients with an absolute indication for a PPI who were not being treated with one. Thus, the purpose of this quality initiative was to determine whether patients with ESKD were appropriately prescribed DAPT (ie, 1-year duration post cardiac event) and PPI therapy who were at high risk of GI bleeding.

## Methods

### Ethics

The proposal was reviewed by the Ottawa Health Science Network Research Ethics Board (OHSN-REB). As per their review, the project was felt to fall within the quality initiative, quality improvement, quality assurance, and/or program evaluation and consequently as per the Tri-Council Policy Statement 2, Article 2.5, review by the OHSN-REB was not required. The project was registered at IQ@TOH Project Registry.

A database was created by The Ottawa Hospital Methods Centre and populated with information collected from the electronic medical record (EMR) of patients being treated at one of the satellite HD units of the Ottawa Hospital. The collected data included age, sex, etiology of ESKD, duration of ESKD, comorbid illnesses and use of antiplatelet agents including aspirin, anticoagulants (including dialysis circuit anticoagulation), corticosteroids, NSAIDs, and PPI.

The patient’s EMR was reviewed for an absolute indication to be treated with a PPI for longer than 4 to 8 weeks including a history of: (1) erosive esophagitis (Grade C or D); (2) Barrett’s esophagus; (3) idiopathic ulcer with a significant GI bleed; (4) NSAID use plus at least one of age > 65 years, prior ulcer, concurrent anticoagulation, antiplatelet, or corticosteroid; (5) verification of need for ongoing DAPT plus at least one of age > 65 years, history of an idiopathic ulcer with a significant GI bleed, concurrent anticoagulation, NSAID, or corticosteroid; (6) Zollinger-Ellison syndrome; and/or (7) esophageal strictures from gastroesophageal reflux disease (GERD).^11,12^ After review, the primary nephrology team was notified by the investigators of this study about any patient with an absolute indication for a PPI such that one can be prescribed if no history of allergy/intolerance and not already on their medication list. We also identified any patients who were taking a PPI without an absolute indication.

The patient’s EMR was also reviewed for the appropriate ongoing use of DAPT. The optimal duration of DAPT therapy is controversial. In the Focused 2018 Update of the Canadian Cardiovascular Society guidelines for the use of antiplatelet therapy, several recommendations were made.^13^ Dual antiplatelet therapy duration if the percutaneous coronary intervention (PCI) was for a non-acute coronary syndrome (ACS) indication was for 6 months but could be prescribed for 3 months to 1 year depending on risk of thrombosis or bleeding (weak recommendation). If the PCI was for ACS, the recommended duration for DAPT was 1 year with extension to 3 years if the treatment was tolerated in the first year and the patient was not at high risk to bleed. Those factors associated with a high risk to bleed of relevance to patients with ESKD included advanced age (>75 years), frailty, anemia (hemoglobin <110 g/dL), and chronic renal failure with a creatinine clearance <40 mL/min. All patients with ESKD have a creatinine clearance <40 mL/min, almost all are anemic, many are frail and are of advanced age; consequently optimal DAPT duration was defined ≤ 1 year. For ESKD patients who were still on DAPT outside the 1-year window, the patient and the primary nephrology team were contacted as appropriate before discontinuing the thienopyridine (clopidogrel, ticlopidine, prasugrel) or ticagrelor. A note was added to each patients’ EMR after the quality improvement review.

### Analysis

The population and outcomes are reported descriptively as follows: (1) mean with standard deviation, or median and interquartile range (IQR) for continuous outcomes; and (2) proportions with 95% confidence intervals for categorical outcomes.

## Results

Of the original 89 patients with ESKD on the satellite HD unit list, 88 were still being treated at The Ottawa Hospital at the time of the chart review in July 2023. The patients were 67.1 (15.8) years of age on average, mostly male (60.2%) and had ESKD for 2.4 (IQR, 1.7-3.8) years (Table 1). The most common causes of ESKD were diabetes mellitus and glomerulonephritis. Approximately 50% of the patients had a history of cardiac disease including 36% with coronary artery disease.

**Table 1.** Demographics of the Satellite Hemodialysis Population.

Age (y)	67.1 (15.8)
Sex (male %)	60.2
Duration of ESKD (M, IQR), y	2.4 (1.7, 3.8)
**Etiology of ESKD (%)**	
Diabetes mellitus	26.1
Glomerulonephritis	28.4
Ischemic	15.9
PKD	2.3
Unknown	5.7
Other	22.7
**Comorbid illnesses (%)**	
Diabetes mellitus	47.0
Malignancy	17.1
Atrial fibrillation	11.4
Congestive heart failure	31.8
Coronary artery disease	36.4
DVT/PTE	5.7

*Note.* ESKD = end-stage kidney disease; M = median; IQR = interquartile range; PKD = polycystic kidney disease; DVT = deep vein thrombosis; PTE = pulmonary thromboembolism.

Forty-four patients were prescribed antiplatelet therapy (4 on DAPT), 1 NSAID, 12 corticosteroids, 7 oral anticoagulants, 2 H2-receptor antagonists, and 39 PPIs. Based on chart review, the most common reason for PPI use was GERD followed closely by corticosteroid use (Table 2). Besides one patient who was prescribed apixaban, all other patients received dialysis circuit anticoagulation with tinzaparin with average dose of 3032 ± 886 (range, 0-5000) units.

**Table 2.** Indications for Proton Pump Inhibitor per Patient.

GERD	13
Prednisone (including previous use)	9
DAPT (including previous use post CABG, post PCI)	6
Started in the ICU	3
Unexplained anemia, drop in hemoglobin	3
Barrett’s esophagus	1
Ischemic colitis	1
Diarrhea	1
Mild gastritis	1
Dysphagia	1
Idiopathic ulcer with bleed	1
Grade B esophagitis	1
Previous NSAID use	1
Anticoagulation	1
Chemotherapy	1
Unknown	1

*Note.* GERD = gastroesophageal reflux; DAPT = dual antiplatelet therapy; CABG = coronary artery bypass grafting; PCI = percutaneous cutaneous intervention; ICU = intensive care unit; NSAID = non-steroidal anti-inflammatory drugs.

Three patients appeared to have been prescribed a PPI for more than one reason: GERD and prednisone, GERD plus dysphagia, prednisone, and aspirin. Seven patients were taking a PPI that was started for a “resolved” indication (post coronary artery bypass graft surgery, post PCI, previous high dose intravenous glucocorticoids, previous NSAID), but the PPI was not discontinued. Only 14% of PPI users had an absolute indication for therapy. One patient had an indication for a PPI (NSAID with advanced age) but had not been prescribed one. After review, the patient’s nephrology team was contacted and the NSAID was discontinued.

Of 4 DAPT users, 3 patients were on DAPT due to coronary artery disease and 1 patient due to coronary artery disease and prior stroke (Table 3). Three patients had a current indication for therapy, whereas 1 patient had a prior indication for DAPT. This patient was treated with DAPT for non-ST elevation myocardial infarction in the setting of severe multivessel disease and he ultimately underwent revascularization with coronary artery bypass graft surgery. He had been on DAPT for over 3 years, but the patient was lost to cardiology follow-up so it had not been re-assessed for discontinuation prior to our chart review. After communicating this information with the patient’s primary nephrology team, the patient was ultimately reduced to single antiplatelet therapy appropriately in review with cardiology recommendation.

**Table 3.** Indications for Dual Antiplatelet Therapy per Patient.

Coronary artery disease	
Coronary artery bypass grafting^a^	2
PCI with drug eluting stent	1
Coronary artery disease with bare metal stent and history of stroke	1

*Note.* CABG = coronary artery bypass grafting; PCI = percutaneous cutaneous intervention.

a One patient who underwent CABG was inappropriately on DAPT.

## Discussion

At the time of this study, only 3% of patients with ESKD treated with HD had a current indication for DAPT. Each of these patients had been prescribed a PPI. One patient prescribed DAPT no longer met the criteria for DAPT use, and after review with their primary nephrology team, the patient was reduced to single antiplatelet therapy. Proton pump inhibitor use was prevalent with most users having relative rather than absolute indications for PPI therapy, similar to our previous study.^10^ Only one patient (1%) with an absolute indication for PPI therapy (NSAID + > 65 years old) had not been prescribed one. Thus, prescribing patterns of DAPT and PPIs are unlikely to be major contributors to the known increased risk of GI bleeding in ESKD patients treated with HD, at least in our center.

The appropriate use of PPIs in patients at high risk for GI bleeds and DAPT therapy duration seen in our study differs compared to studies in other patient populations. In a large Korean database, 50% of patients at high risk to experience a GI bleed post myocardial infarction treated with DAPT were prescribed a PPI.^14^ Furthermore, in regard to antiplatelet therapy, Ardoino et al^15^ found that there was a high degree of inappropriate prescription of antiplatelet therapy in acutely hospitalized older adults for both primary and secondary prevention of cardio- or cerebrovascular disease, with over 50% of study patients inappropriately prescribed for primary prevention.

Routine periodic medication reconciliation performed within our dialysis unit may have been a key factor in the largely appropriate prescribing patterns seen in our study. The policy at our center is for the EMR to be updated every 3 months by nurses performing the medication reconciliation with patients using pill bottles or pharmacy records, and subsequently verified by the most responsible physician. We suggest that dialysis units may benefit from having policies in place such as scheduled periodic medication reviews by the nephrology team to limit prolonged prescription of DAPT and ensuring the use of PPIs in individuals who are deemed high risk to experience a GI bleed.

Several issues require further study. About 10% of patients in our study were currently being treated with corticosteroids; most were receiving a PPI for this indication. In a 2010 survey of 360 physicians from different specialties, 82% considered corticosteroids to be ulcerogenic.^16^ In a systematic review and meta-analysis that included more than 30 000 patients being treated with corticosteroids, 804 patients experienced a GI bleed or perforation (2.9% and 2.0% for corticosteroids and placebo).^17^ Corticosteroids increased the risk of GI bleeding or perforation by 40% (odds ratio [OR] = 1.43, 95% CI = 1.22-1.66). However, the risk was statistically increased only for hospitalized patients and not those followed in ambulatory care. Patients with ESKD as a subgroup were not examined. It is unclear whether the comorbid burden, frequent hospitalizations, and use of treatment-related anticoagulants in the ESKD population might pose an increased risk of bleeding with corticosteroids.

In the guidelines, one of the absolute indications for prescribing a PPI is an antiplatelet agent with one other history of ulcer, concomitant anticoagulation, or NSAID.^18^ Although concomitant anticoagulation refers to the use of warfarin or direct oral anticoagulants, the impact of thrice weekly systemic anticoagulation for patients treated with HD with and without antiplatelet agents is not clear. In a study using Taiwan’s National Health Insurance program, several different models (including propensity matched) were created to explore the risk of hospitalization for GI bleeding in patients treated with HD compared with peritoneal dialysis; all showed an increased risk of bleeding in patients treated with HD.^19^

Our study has several limitations. The study population included only one satellite unit of The Ottawa Hospital which has a relatively small number of patients and likely limits the generalizability of our results. In addition, the medication lists were taken from the EMR and not verified with patient interview at the time of data collection. Although periodic medication reviews are performed by the nurses in the HD unit, in our previous study, we did find about a 10% discrepancy between the use of PPIs by the medication list and patient interview.^10^

In the future, large-scale administrative databases may be used to determine whether outpatient corticosteroid use is associated with an increased risk of GI bleeding in patients with ESKD compared with the general population after adjusting for comorbid illnesses. The potential importance of duration of DAPT therapy and use/dose of HD treatment anticoagulation as risk factors for GI bleeding are unlikely to be clarified using administrative datasets as over-the-counter medications (eg, aspirin) and hospital administered medications (eg, tinzaparin/heparin) are not captured.

In summary, we did not find that the inappropriate use of DAPT or lack of PPI use in patients at high risk for upper GI bleeds were likely contributors to the known increased risk of GI bleeding in patients with ESKD treated with HD. Periodic review of medications within dialysis units may help to ensure appropriate medication prescription in patients and rectify situations whereby PPI and DAPT have not been appropriately prescribed.
